# Extrusion of Femtosecond Laser-Implanted Intrastromal Corneal Ring Segments in Keratoconic Eyes: Prevalence, Risk Factors, and Clinical Outcomes

**DOI:** 10.1155/2020/8704219

**Published:** 2020-03-28

**Authors:** Amr Mounir, Mahmoud Mohamed Farouk, Marwa Mahmoud Abdellah, Engy Mohamed Mostafa

**Affiliations:** Department of Ophthalmology, Sohag Faculty of Medicine, Sohag University, Sohag 82524, Egypt

## Abstract

**Purpose:**

To evaluate the prevalence, possible risk factors, and clinical results of femtosecond laser implanted intrastromal corneal ring segment (ICRS) extrusion in keratoconic eyes. *Patients and Methods*. This is a retrospective observational study evaluating 333 eyes of 269 patients who were subjected to femtosecond laser-implanted Keraring ICRS in the Sohag Refractive Center, Sohag, Egypt, from January 2014 to January 2019. The study included eyes with channels created by a femtosecond laser (60 kHz IntraLase femtosecond system; Advanced Medical Optics, Santa Ana, California, USA) with implantation of Keraring intrastromal corneal ring segments (Mediphacos, Belo Horizonte, Brazil). Patient data and causes of Keraring extrusions were identified as being those rings that migrated or showed melting of the cornea with no other reason which required segment removal.

**Results:**

Seven eyes were found to fit the criteria of ring extrusion (2.1%) out of the 333 eyes which had Keraring implantation. All extruded rings were from patients with keratoconus grade 3, with eccentric cones, and with femtosecond creation of the tunnel. Four eyes belonging to 3 patients (57.1%) had a history of vernal Keratoconjunctivitis, yet they did not show signs of activity at the time of implantation. They reported excessive rubbing just before they presented with conjunctival hyperemia and foreign body sensation. Five eyes (71.4%) showed chronic sun exposure. The mean minimal corneal thickness was 401.85 *μ*m (range 384–420 *μ*m), while the mean maximum keratometry was 61 D (range 55.18–68.96 D). Most of the extruded rings had large arcs. Six eyes had crosslinking (CXL) at the same session of the Keraring implantation. The simultaneous CXL treatment is considered as a possible significant risk factor for ring extrusion.

**Conclusion:**

ICRS is an effective reversible option for patients with keratoconus who are intolerant to hard contact lenses, yet the choice of cases and ring segments is mandatory for satisfactory results. Moreover, meticulous history taking and examination reduces the incidence of complications including extrusion.

## 1. Introduction

Implantation of intrastromal corneal ring segments (ICRS) is an effective and reversible refractive technique for keratoconus management [1]. Different types of incomplete rings are used in the management of keratoconus: Intacs (AdditionTechnology, Inc.), Ferrara ring (Ferrara Ophthalmics Ltd.), and Keraring (Mediphacos Ltd.) [2].

The reversibility of ICRS implantation has been confirmed in keratoconic eyes and poses a major advantage as the technique of implantation does not involve tissue removal [3].

Despite the positive reports of ICRS implantation in managing corneal ectatic disease, some complications have been reported such as incomplete tunnel creation, anterior or posterior corneal perforation, epithelial defects, and segment extrusion [4–9].

Ring segment extrusion is one of the late complications of ICRS implantation, leading to ring explantation [7]. Different causes have been supposed as an etiology including ring segment migration and corneal melting which precede total ring segment extrusion [10].

Ring implantation by mechanical tunnel creation showed more common extrusion than tunnel creation performed by the femtosecond laser (FS) [11]. Femtosecond laser-created corneal tunnels are an effective procedure to avoid late ring segment extrusion as it made the corneal ring implantation procedure faster, easier, and safer with precise depth of implantation [12].

The aim of our study is to evaluate the prevalence, possible risk factors, and clinical results of the femtosecond laser-implanted ICRS extrusion in keratoconic eyes.

## 2. Patients and Methods

This is a retrospective observational study evaluating 333 eyes of 269 patients who were implanted with Keraring ICRS in the Sohag Refractive Center, Sohag, Egypt, from January 2014 to January 2019. This study followed the tenets of Declaration of Helsinki, and Ethical Board Committee approval from the institution was obtained.

Patient data and causes of Keraring extrusions were identified as being those rings that migrated or showed melting of the cornea with no other obvious reason which required segment removal. Exclusion criteria included eyes with elective explantation for refractive dissatisfaction, infective or noninfective keratitis, or visual disturbance and ring implantation indications other than keratoconus and mechanical creation of the ring tunnel.

The study included eyes with channels created by a femtosecond laser (60 kHz IntraLase femtosecond system; Advanced Medical Optics, Santa Ana, California, USA) with implantation of Keraring intrastromal corneal ring segments (Mediphacos, Belo Horizonte, Brazil).

All patients had 1 or 2 Keraring ICRS implanted for the treatment of keratoconus (grade 1, 2, and 3 according to Amsler-Krumeich classification) following the Keraring nomogram rules. It was taken into consideration that the central cornea was clear and age range was between 18 and 40 years. The minimum corneal thickness at the implantation site was 350 *μ*m at the thinnest corneal point and at least 400 *μ*m at the incision site. All cases underwent full ophthalmological evaluation including uncorrected visual acuity (UCVA), best spectacle corrected visual acuity (BSCVA), slit-lamp biomicroscopy (dry eye grading) [13], manifest and cycloplegic refraction, and corneal tomography using Sirius Scheimpflug placido topography (Costruzione Strumenti Oftalmici, Florence, Italy).

### 2.1. Primary Implantation Procedure

The surgical procedures were performed by the two surgeons involved in this study under topical anesthesia and complete aseptic measures. The procedure was initiated by marking a reference point for centration (Purkinje reflex). Continuous circular stromal tunnel was created as recommended by ring manufacturers and previous studies [8, 14]. The incision axis was planned on the axis of the steepest keratometric reading at an 80% depth of the thinnest location as determined by optical and ultrasound pachymetry with inner diameter 5.00 mm, outer diameter 5.90 mm, entry cut length 1.40 mm, and entry cut thickness 1 mm. Incision was created at the steepest axis, with ring energy 1.9 mJ. After ring insertion, a soft bandage contact lens was applied. Postoperatively, topical moxifloxacin and prednisolone acetate eyedrops were used with topical preservative-free lubricants.

### 2.2. Explantation Procedure

Explantation of extruded ring segments was done under complete aseptic conditions. The extruded side of the segment was pulled out with corneal forceps. A Sinskey hook was introduced to grab the segment at its distal end near the wound until completely pulled out of the tunnel. In some cases where melting was established, the ring was removed from the area it protruded from. No sutures were used. A soft bandage contact was applied to allow healing of the empty tunnel. After all procedures, moxifloxacin and dexamethasone eyedrops were used 4 times a day along with lubricant eyedrops 5 times a day for 2 weeks. The analysis comprised data from pre- and postoperative months. The rate and causes of extrusions were reported and analyzed throughout the six years. Slit-lamp examination to evaluate the ICRS position and corneal integrity after explantation was performed. Subsequent postextrusion follow-up included UCVA, BCVA, manifest refraction, slit-lamp evaluation, and corneal tomography.

### 2.3. Corneal Crosslinking (CXL) Procedure

Eyes that showed progression over follow-up periods were subjected to crosslinking. Inclusion criteria for accelerated epithelium on CXL had thinnest corneal thickness of 400 *μ*m. CXL was performed following the ring implantation in the same session. CXL steps were as follows: Dextran-free hypo-osmolar riboflavin drops which contain benzalkonium chloride to enhance epithelium permeability (ParaCel, Avedro, Waltham, Massachusetts, USA) was applied every 1.5 min for 4.5 min, followed by benzalkonium chloride-free riboflavin drops (VibexXtra, Avedro, Waltham, Massachusetts, USA) every 1.5 min for 6 min. Then, accelerated CXL was used for 2 min and 40 seconds in a pulsed mode (2 seconds on/1 second off) (KXL accelerated CXL System, Avedro, Waltham, Massachusetts, USA). The power used was 45 mW/cm^2^ with a total energy radiated of 7.2 J/cm^2^. A contact lens was then applied for one week and eyedrops were prescribed for 4 weeks in the form of artificial tears, steroids, and antibiotics.

## 3. Statistical Analysis

Statistical analysis was performed using SPSS for Windows software (version 15.0.1, SPSS, Inc.). Student's *t* test for paired data was used to compare the preoperative and postoperative data (keratometry, sphere, etc.). The Wilcoxon rank test was used for comparing the rate of extrusions throughout the five years.

## 4. Results

**Figure 1:**
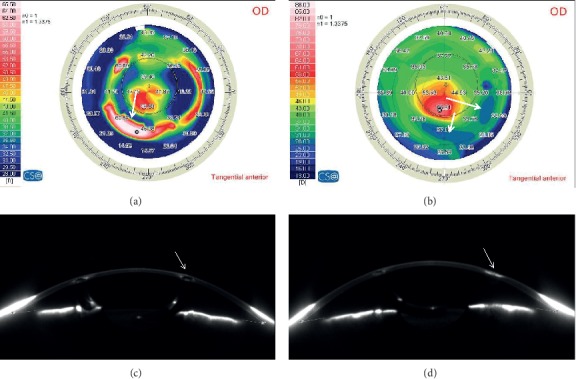
(a) Keratometry map before Keraring extrusion; the arrows pointing to the steepening correspond to the Kerarings. (b) Keratometry map after ring extrusion; the arrows pointing to the flattening correspond to the extruded rings. (c) Scheimpflug densitometry of implanted Kerarings. (d) Scheimpflug densitometry showing the scar at the site of extruded Keraring.

Seven eyes were found to fit the criteria of ring extrusion (2.1%) out of the 333 eyes which had Keraring implantation. The mean age of the patients in the extrusion group was 20.27 years ± 4.75 (SD) (range from 18 to 25 years), while mean age in the control group was 21.7 ± 5.3 with no statistical difference between groups. Comparison between the refractive data between eyes with extrusion and eyes with no extrusion are represented in Table 1. There was no statistical difference on comparing the prering implantation refractive data in both groups as well as postimplantation data. Analysis of the refractive data of the seven eyes before ring extrusion and after extrusion is demonstrated in Table 2. The refractive effect of the rings was reversed as they were removed (Figure 1).

Table 3 summarizes the possible risk factors in the ring extruded cases. There was no history of trauma, and no report of infection was found. All extruded rings were from patients with keratoconus grade 3 with eccentric cones with femtosecond creation of the tunnel. Females showed higher contribution to these cases (6 out of the 7 eyes) (85.7%). Four eyes belonging to 3 patients (57.1%) had a history of vernal keratoconjunctivitis, yet they did not show signs of activity at the time of implantation. They reported excessive rubbing just before they presented with conjunctival hyperemia and foreign body sensation. Five eyes (71.4%) showed chronic sun exposure (more than 6 hours a day). All eyes suffered from dry eye disease with grades ranging from 2 to 3 in severity. The mean thinnest corneal thickness was 401.85 *μ*m (range from 384 to 420 *μ*m) while the mean maximum keratometry was 61 D (range 55.18–68.96 D). The mean time interval between Keraring implantation and extrusion was 6.6 months (range 3–12 months). Five eyes out of the seven had 2 rings implanted in the same eye. Also, five eyes had thick rings implanted 300/250, and the other 2 eyes had rings with a thickness of 250/250. Most of the extruded rings had large arcs. Six eyes had crosslinking (CXL) at the same session of the Keraring implantation.

Table 4 shows the difference between the group with extruded rings and the nonextruded group. Statistically significant difference was found in the following parameters: dry eye, stage of KC, site of the cone, eyes implanted with two rings, thicker rings, increasing ring arc, and simultaneous CXL session.

**Figure 2:**
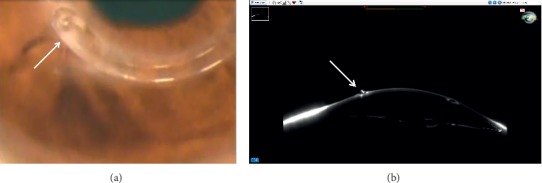
(a) Extruded Keraring. (b) Scheimpflug densitometry showing Keraring extrusion.

**Figure 3:**
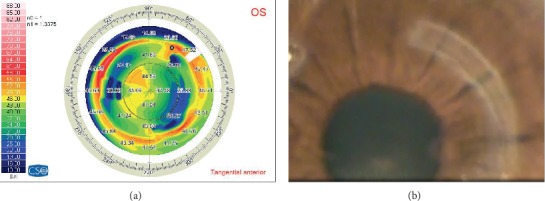
(a) Keratometry map after Keraring extrusion showing corresponding corneal flattening. (b) Corneal scar corresponding to the site of extruded Kerarings.

Ring migration, which is considered an early stage before extrusion, was noted in 4 eyes (57.1%), while corneal melting was found in 5 eyes (2 eyes with ring migration) (Figure 2). In the eyes with corneal melting, the edge of the ring would protrude through the stroma and not necessarily through the primary incision site. Two eyes showed corneal opacification after ring removal (Figure 3).

Eyes with extruded rings were no further managed by reimplantation of ICRS; 4 eyes were left with no further intervention and the rest proceeded to penetrate keratoplasty.

## 5. Discussion

In the current study, we are reporting the cases with Keraring ICRS extrusion in a case series of ICRS implantation. We were trying to detect potential risk factors and contributing parameters for ring extrusion to be taken into account to decrease such complication.

The rate of ring extrusion in the current study is 2.1%, while others reported 6.4% [10], 19.7% [15], and 30% [7]. The higher incidence can be explained that these rings were implanted in tunnels created by manual dissection. Ferrer et al. [16] reported ICRS extrusion in 28 eyes out of 250 (10.9%), where they found macrophages with extracellular matrix (ECM), cells, collagen, and exopolysaccharide in 68.8% of both ends of the extruded segments using scanning electron microscopy.

Also, in a study by Monteiro et al. [17], they compared the incidence of complications between manual and femtosecond laser-assisted surgery for ICRS implantation and found that there were complications in 18.11% of eyes of the manual group while in the femtosecond laser only 3.6% of eyes. In the manual group, ICRS spontaneous extrusion occurred in 5.66%.

The creation of the channels in our study was done by the femtosecond laser which rules out the uneven depth creation by mechanical dissectors which is reported by other studies. The shortest time that elapsed from the time of implantation till the time of extrusion was 3 months and the longest was 12 months. A study by Oatts et al. reported late extrusion as late as 20 years [18]. The interval elapsing between implantation and extrusion is not the same in all cases, and the mechanism remains elusive. In the seven eyes reported, no obvious local triggering effect and only spontaneous extrusion occurred. One attributing mechanism that might have a role is severe stromal thinning over the implanted rings despite being implanted at an appropriate depth. We also concluded that thicker rings implanted in thinner corneas might play a role in extrusion. The creation of the channels in our study was done by the femtosecond laser which rules out the uneven depth creation by mechanical dissectors which is reported by other studies.

CXL was noted to be associated with all seven cases reported. Six eyes had CXL in the same session which might draw the conclusion that CXL might play a role in the migration and later extrusion of the rings. On searching the literature, no studies were found that link CXL to such a complication which necessitates further research.

We also found that dry eye disease (DED) could also play a role as it was reported to be more prevalent in keratoconus [19]. This might be attributed to the alteration in diffusion of metabolites due to the formation of dellen over the rings along with biomechanical stress [18].

We understand that our study has limitation as there is lack of histologic studies of the explanted rings which might have aided in understanding the potential biomechanical cause. There are histologic reports of corneas removed for penetrating keratoplasty documenting the presence of hypoplasia of the epithelium surrounding the channel with decreased keratocytes density above and below the tunnel and collagen IV synthesis even up to 27 months following implantation. These findings proved to be reversible after 6 months [20].

Four eyes out of the seven had a history of chronic atopic keratoconjunctivitis (AKC) which has been linked to keratoconus [21]. One possible explanation is that the implanted rings can induce chronic foreign body sensation which triggers an inflammatory reaction with the release of MMP2 and MMP9 in the tear film. These enzymes play a role in degrading the basement membrane of the corneal epithelium [22]. This also can explain why not all rings extrude through the incision site and extrude through corneal melt.

Ring migration in our case series occurred more in long arc thick ring segments just before extrusion. In our experience, if a ring migrated in the range of 0.5 mm near the incision site, it should be followed closely with a trial of pushing it back into the tunnel for 2 mm to avoid dislodging if further movement occurred. Yet, if the movement recurred, explantation would be recommended.

In conclusion, ICRS use in the cases of KC is by far a viable option for patients who are intolerant to hard contact lenses, yet the choice of cases, meticulous preoperative evaluation, and timing of CXL are variables to be considered and well chosen for satisfactory long-term results.

## Figures and Tables

**Table 1:** Refractive changes in all eyes comparing the eyes with extrusion with eyes of no extrusion and pre- and postimplantation of ICRS.

	Eyes with ring extrusion (*n* = 7)		Eyes with no extrusion (*n* = 326)		*P* value of preimplantation values in both groups	*P* value of postimplantation values in both groups
	Preimplantation	Postimplantation	Preimplantation	Postimplantation		
Sphere	7.1 ± 3.2	4.8 ± 2.1	8.2 ± 2.8	5.2 ± 2.1	0.53	0.44
Cylinder	6.5 ± 1.7	3.3 ± 1.9	6.1 ± 2.1	4.1 ± 1.4	0.35	0.43
UCVA (logMar)	1.2	0.8	1.2	0.7	0.93	0.81
BSCVA (logMar)	0.8	0.6	0.8	0.6	0.32	0.27
Thinnest CT (*μ*m)	404	401	405	402	0.63	0.56
Max *K* (D)	61.1	56.3	61.7	57.2	0.23	0.33

UCVA, uncorrected visual acuity; BSCVA, best spectacle corrected visual acuity; CT, corneal thickness; *K*, keratometry.

**Table 2:** Refractive changes after extrusion.

	Prering extrusion	Postring extrusion
UCVA (logMar)	0.8	1.0
BSCVA (logMar)	0.4	0.8
Thinnest CT (*μ*m)	404	401
Max *K* (D)	56	61

UCVA, uncorrected visual acuity; BSCVA, best spectacle corrected visual acuity; CT, corneal thickness; *K*, keratometry.

**Table 3:** Presumable risk factors in the ring extruded cases.

	Patient factors					Topography factors				Keraring factors				
Patient #	Age	Sex	Atopic association	Sun exposure	Dry eye severity level	Stage of KC	*K* max	Cone position	Minimal CT	Number of rings	Extrusion after mns	Ring thickness	Ring arc	CXL timing
1	25	F	+	−	2	3	58.57	Eccenteric	420	2	6	300–250	160–160	Same session
2	18	F	−	+	2	3	59.7	Eccenteric	410	2	3	300–300	160–160	Same session
3	18	F	−	+	3	3	61.22	Eccenteric	390	2	5	250–250	160–160	Same session
4	23	F	−	−	2	3	62.92	Eccenteric	402	2	8	300–300	210–90	Same session
5	20	F	+	+	3	3	55.18	Eccenteric	395	2	12	300–250	210–90	Same session
6	21	F	−	+	3	3	60.57	Eccenteric	384	2	12	300–250	160–160	Same session
7	19	M	+	+	3	3	68.96	Eccenteric	412	2	7	300–300	160–90	Sequential

F, female; M, male; KC, keratoconus; *K*, keratometry; CT, corneal thickness; CXL, crosslinking.

**Table 4:** Comparison of presumable risk factors between eyes with and without ring extrusion.

	Eyes with ring extrusion (*n* = 7)	Eyes with no extrusion (*n* = 326)	*P* value
Atopic association	3 (42.9%)	144 (44.1%)	0.33
Chronic sun exposure	5 (71.4%)	216 (66.3%)	0.085
Dry eye severity level			
0	—	48 (14.7%)	
1	—	50 (15.3%)	
2	3 (42.9%)	120 (36.8%)	0.044
3	4 (57.1%)	108 (33.1%)	0.03
Stage of KC			
Stage 1	—	84 (25.8%)	
Stage 2	—	112 (34.4%)	
Stage 3	7 (100%)	130 (39.9%)	0.02
*K* max			
<55 D	—	84 (25.8%)	
55–62 D	5 (71.4%)	144 (44.2%)	0.052
>62 D	2 (28.6%)	98 (30.1%)	0.063
Cone position			
Central	—	219 (67.2%)	
Eccentric	7 (100%)	107 (32.8%)	0.001
Number of rings			
One ring	—	194 (59.5%)	0.82
Two rings	7 (100%)	132 (40.5%)	0.001
Ring thickness			
300	8 (57.1%)	128 (27.9%)	0.03
250	6 (42.8%)	219 (47.8%)	0.087
200	—	111 (24.2%)	
Ring arc			
90	3 (21.4%)	105 (22.9%)	0.33
160	9 (64.2%)	288 (62.9%)	0.05
210	2 (14.2%)	65 (14.2%)	0.063
Same session CXL	6 (85.7%)	241 (73.9%)	0.01

## Data Availability

The data used to support the findings of this study are available from the corresponding author upon request.
